# Skin sensitivity to capsaicin, perceived stress and burn out among patients with building-related symptoms

**DOI:** 10.1007/s00420-020-01647-x

**Published:** 2021-01-10

**Authors:** Bo Glas, Anna-Sara Claeson

**Affiliations:** 1 Department of Clinical Medicine and Public Health, Umeå University, Umea, Sweden; 2 Department of Psychology, Umeå University, Umea, Sweden

**Keywords:** Sick building syndrome, Skin, Capsaicin, TRPV1, Perceived stress burneout

## Abstract

**Objective:**

The mechanisms behind building-related symptoms have remained unknown despite many years of research. It is known that environmental and psychosocial factors are of importance. Some receptors in the Transient Receptor Potential family elicit the same symptoms when stimulated, as reported by those suffering from building-related symptoms. The aim of this study was to compare capsaicin sensitivity between people with and without skin symptoms. A second aim was to investigate perceived stress among individuals with different levels of capsaicin sensitivity.

**Methods:**

People referred to an occupational health care unit and judged to have building-related symptoms by a physician answered a questionnaire regarding their symptoms. Solutions with different capsaicin concentrations were applied to participants’ nasolabial folds. Self-reported stress and burnout were assessed using two questionnaires: the Perceived Stress Scale (PSS) and the Shirom-Melamed Burnout Questionnaire (SMBQ).

**Results:**

We found that people who reported facial erythema or itching, stinging, tight or burning facial skin were more sensitive than those without symptoms and similarities with Sensitive Skin are discussed. We also found that participants who reacted to the lowest capsaicin concentrations scored significantly higher on scales for stress and burnout.

**Conclusions:**

We found associations between sensitivity to capsaicin and skin symptoms among people with building-related symptoms, as well as associations between capsaicin sensitivity and perceived stress/burnout.

## Introduction

The occurrence of building-related symptoms (BRS), that some people perceive when they stay in certain buildings has been known for a long time, as well as different risk factors like low ventilation (Fisk et al. 2009; Ige et al. 2018), dampness and mould (Sauni et al. 2015) and inadequate cleaning (Kemp et al. 2004; Skulberg et al. 2004). All these factors affect indoor air quality and chemical content of the air, but the mechanisms resulting in symptoms remain unknown. Commonly reported symptoms are skin symptoms (e.g. dryness, flushing, burning, itching, stinging sensations etc.), symptoms from airways (e.g. irritated/stuffy nose, cough, hoarseness, dry throat), eyes (e.g. dry/irritated/itching eyes) and general symptoms like tiredness and headache (Norback 2009; World Health Organization 1983). The TRPV1 receptor (Transient Receptor Potential Vanilloid type 1) is expressed in the skin and gives rise to flushing, burning, itching, stinging sensations when stimulated (Deng and Li 2005; Gouin et al. 2017; Jourdain et al. 2005) i.e. the same symptoms as reported by those with BRS. It belongs to a group of receptors, Transient Receptor Potential (TRP) ion channels that convert chemical, physical, and mechanical stimuli to electrical or chemical signals (O'Connor and Adams 2010). TRPV1 is stimulated by a number of different chemicals like capsaicin, temperatures higher than 43 °C, low/high pH, UV-light and endogenous signal substances. In the skin, TRPV1 is mainly expressed by free nerve endings and neurons but also by several types of cells like keratinocytes and endothelial cells. The receptor is engaged in a number of processes in the skin like neurogenic inflammation and perception of pain and itching (Gouin et al. 2017; Moore et al. 2018). Reactions to TRPV1 agonists can be used as an indicator of skin sensitivity (Jourdain et al. 2005).

If air contaminants are to induce skin symptoms, they have to enter the viable parts of the skin. This consists of several “layers”. The outermost part, the epidermis, consists mainly of keratinocytes, but there are also other types of cells and structures, like nerve endings. The epidermis is continuously renewed when new keratinocytes are formed in the basal cell layer and move toward the surface. During the process, the cells are emptied of cytoplasm and the nucleus disappears, forming corneocytes and finally only the cornified envelope remains, mainly made up of keratins. This layer, stratum corneum (SC), is the outer shell protecting the body from the extrinsic environment, from dehydration which helps maintain homeostasis (Candi et al. 2005). The intercellular space between corneocytes consists of a complex mixture of lipids and water binding substances. It is possible for small lipophilic chemicals to enter the skin through diffusion between corneocytes, though it is a slow process. An impaired SC makes it easier for chemicals to pass the protective layer (Kezic and Nielsen 2009). Symptoms related to the skin are commonly reported in BRS, but there is currently a lack of knowledge about the mechanisms behind the symptoms.

The association between BRS and stress has been identified in a number of studies and perceived stress is often put forward as a risk factor for BRS (Azuma et al. 2015; Bakke et al. 2007; Lu et al. 2018; Marmot et al. 2006). Further, psychological stress influences the skin by influencing barrier function and wound healing (Choi et al. 2005; Hunter et al. 2015; Jafferany and Patel 2019). Psychological stress may affect the ability of SC to protect epidermis from air contaminants and which make people more susceptible to external noxious stimuli. The TRPV1 receptor may play a role in this and in a study on mice, Zheng et al. (2015) found that chronic stress increased the expression of TRPV1 in a subpopulation of nociceptive neurons in dorsal root ganglia, which affected the pain response. This effect of stress in stressed rats on levels of TRPV1 in dorsal root ganglia, paw plantar skin and pain sensitivity was also observed by Ma et al. (2019). The effect was abolished by subcutaneous injections of capsazepine, a TRPV1 antagonist. In another study, Wang et al. (2017) found that TRPV1 regulates the stress response in the brain. The possible effects of stress on epidermis and the association to symptoms mediated by TRPV1 makes the receptor to an interesting object to study as a candidate to mediate building-related skin symptoms.

The main aim of this study was to investigate if individuals with BRS who report skin symptoms are more sensitive to capsaicin than those who do not report skin symptoms. The second aim was to investigate if individuals with high sensitivity to capsaicin report higher levels of stress (both short term and long term) than individuals with low capsaicin sensitivity.

## Materials and methods

### Participants

Participants in the study worked at Umeå University Hospital, Sweden. They were recruited from personnel with suspected BRS referred to the occupational health care clinic. Individuals that had symptoms that were judged to be due to the indoor environment by a physician were asked to participate.

If they agreed, they were asked to fill out a questionnaire that included questions pertaining to demographics and physician-based diagnoses. The questionnaire also included questions on symptoms related to BRS, categorized as general symptoms (headache, concentration difficulties, tiredness, heavy-headedness), mucous membrane symptoms (cough, throat irritation/hoarseness, nasal congestion/discharge, excessive mucous production, nasal mucosa irritation/dryness, eye irritation, dry eyes) and skin symptoms (facial itching/stinging/tightness/heat, facial redness, dry facial skin, body itching). These symptoms have been listed by the World Health organization to be of importance for building-related health issues (1983) and have been used in earlier studies to identify cases with building-related intolerance (Edvardsson et al. 2008; Glas et al. 2015). The symptoms were rated on a scale including the following alternatives “yes, every week”, “Yes, sometimes” and “No, never”. The reported skin symptoms were then used to identify two groups, one with skin symptoms, were individuals that reported to have skin symptoms often or sometimes were included, and a group with no symptoms were participants reporting no skin symptoms were included (*n* = 14).

Besides the medical examination, to be included in the study the participants had to report to have at least one symptom from either skin or from the mucous membranes often (“yes every week”). They also had to answer yes to the question “Do you think that you have or have had problems/symptoms caused by poor indoor environment in your workplace?” (e.g. self-reported BRS). Further, in order to be included, they should not be on sick leave. Other exclusion criteria were smoking, pregnancy, cancer treatment or change of working place after the visit at the occupational health care clinic.

The study was performed between November 2015–June 2016 and October 2016–March 2017.

There was a group of participants that had been medically examined about 1 year earlier (December 2014–April 2015) and they were invited to participate in the study. These were 42 (former patients) and the number of participants recruited during the study period was 23 (new patients). In total, 65 people were included in the study, 12 men and 53 women between 24 and 66 years old, with a mean age of 45.5 (± 10.9) years.

### Capsaicin test

During the participants’ visit to the occupational health care clinic trained nurses performed a capsaicin test of skin neurosensitivity according to Jourdain (2005). Capsaicin > 95%, article number M2028 from Sigma-Aldrich to 95% ethanol, analytical grade, from Solveco, Sweden, was used. Capsaicin was first dissolved in 95% Ethanol and then prepared in five dilution steps ($\sqrt{10}$) between 3.16 × 10^–3^% and 3.16 × 10^–5^% in 10% ethanol. The test procedure was as follow: Participants’ nasolabial folds were first cleaned with 10% ethanol to let them get use to the feeling. Then the most diluted preparation of capsaicin was topically applied on one side with a cotton swab (17 ± 2 µl) together with 10% ethanol in the other fold, as control. If the participant did not feel a difference between the two sides within 3 min the next concentration was applied. The detection threshold was set to the concentration when participants identified a difference between capsaicin and ethanol and the sensation was experienced for more than 30 s. The side for application of capsaicin and ethanol was changed between each step and participants did not know on what side capsaicin was applied (Jourdain et al. 2005). All tests were performed by the same trained personal.

### Questionnaire

To investigate different aspects of stress, two different questionnaire instruments were used: Perceived Stress Scale (PSS) and Shirom-Melamed Burnout Questionnaire (SMBQ).

The Swedish version of the PSS-10 was used as a measure of short-term perceived stress (Cohen 1988; Nordin and Nordin 2013). The PSS-10 consists of ten items related to perceived stress in everyday life during the last month and the scale ranges from 0 to 40 were a high score indicate a high stress level. The PSS-10 have good reliability and construct validity (Nordin and Nordin 2013).

The SMBQ (Melamed et al. 1992) was used to measure different aspects of mental and physical exhaustion (burnout or long-term stress). This 22-item questionnaire is rated on a 7-point Likert scale from 1 (almost never) to 7 (almost always). The total score is calculated as a mean across all items and higher score indicates more mental and physical exhaustion (e.g. long-term stress). The questionnaire has good construct validity and reliability (Grossi et al. 2003).

### Procedure and statistical methods

Average capsaicin sensitivity (sensitivity defined as detected concentration) were calculated by using the number of the concentration (numbers 1–6 in Table 1) that each participant reacted to. Comparisons of capsaicin sensitivity between participants with and without different skin symptoms were performed using the Mann–Whitney *U*-test (Table 2).

**Table 1:** Denotation of sensitivity, number, sex and age (years) of participants that reacted to each concentration of capsaicin

Sensitivity	Low		Medium		High	
	1	2	3	4	5	6
Detected capsaicin concentration (%)	Did not react	3.16 × 10^–3^	1 × 10^–3^	3.16 × 10^–4^	1 × 10^–4^	3.16 × 10^–5^
Number men/women	6/18	2/12	1/8	0/6	1/7	2/2
Age (SD)	45.7 (10.5)	45.4 (9.65)	44.6 (13.8)	47.5 (11.4)	45.9 (12.6)	44.5 (12.9)

**Table 2:** Comparison of detected concentration groups for capsaicin for participants reporting a symptom often or sometimes with those not having the symptom during the last month (number of participants within brackets)

Symptom	Average capsaicin detected concentrations for participants reporting		
	Skin symptom	No symptom	*p*
Dry facial skin	4.3 (48)	4.9 (17)	0.16
Facial erythema	4.1 (38)	4.9 (27)	0.039*
Itching, stinging, tight or burning facial skin	4.0 (39)	5.0 (26)	0.003*
Body itch	4.3 (31)	4.5 (34)	0.39

Sensitivity for groups is compared with Mann–Whitney *U*-test

*p < 0.05

Based on their sensitivity to capsaicin, the participants were divided into three groups. Those who did not react or reacted to 3.16 × 10^–3^% were considered to have low sensitivity. Those who reacted to 1 × 10^–3^ or 3.16 × 10^–4^% medium sensitivity and those who reacted to 1 × 10^–4^ or 3.16 × 10^–5^% were considered to have high sensitivity. Differences in PSS and SMBQ between the three groups were compared by one-way analyses of variance (ANOVA) followed by post hoc Tukey’s HSD-test. The level of significance was set to *p* = 0.05.

Comparisons of PSS and SMBQ for men/women and new/former patients were performed by *t* test. For statistical calculations IBM SPSS Statistics for Windows, Version 25.0. IBM Corp. Released 2017. Armonk, NY, was used.

## Results

Out of the 65 participants with BRS included in the study, 41 reacted to capsaicin and 24 did not react to any of the prepared concentrations. The number of participants who reacted to each capsaicin concentration, their average age and sex is shown in Table 1. Participants who reported to have itching/stinging/tight/burning facial skin and those with facial erythema were more sensitive to capsaicin than those without symptoms. There was no significant difference between the groups for dry facial skin or body itch (Table 2).

The difference was significant for those with itching/stinging/tight/burning skin and those with facial erythema. Nine participants reported having body itching often and their average detected concentration for capsaicin was 3.3 compared with 4.6 for those having the symptom sometimes or never (*p* = 0.020).

**Fig. 1:**
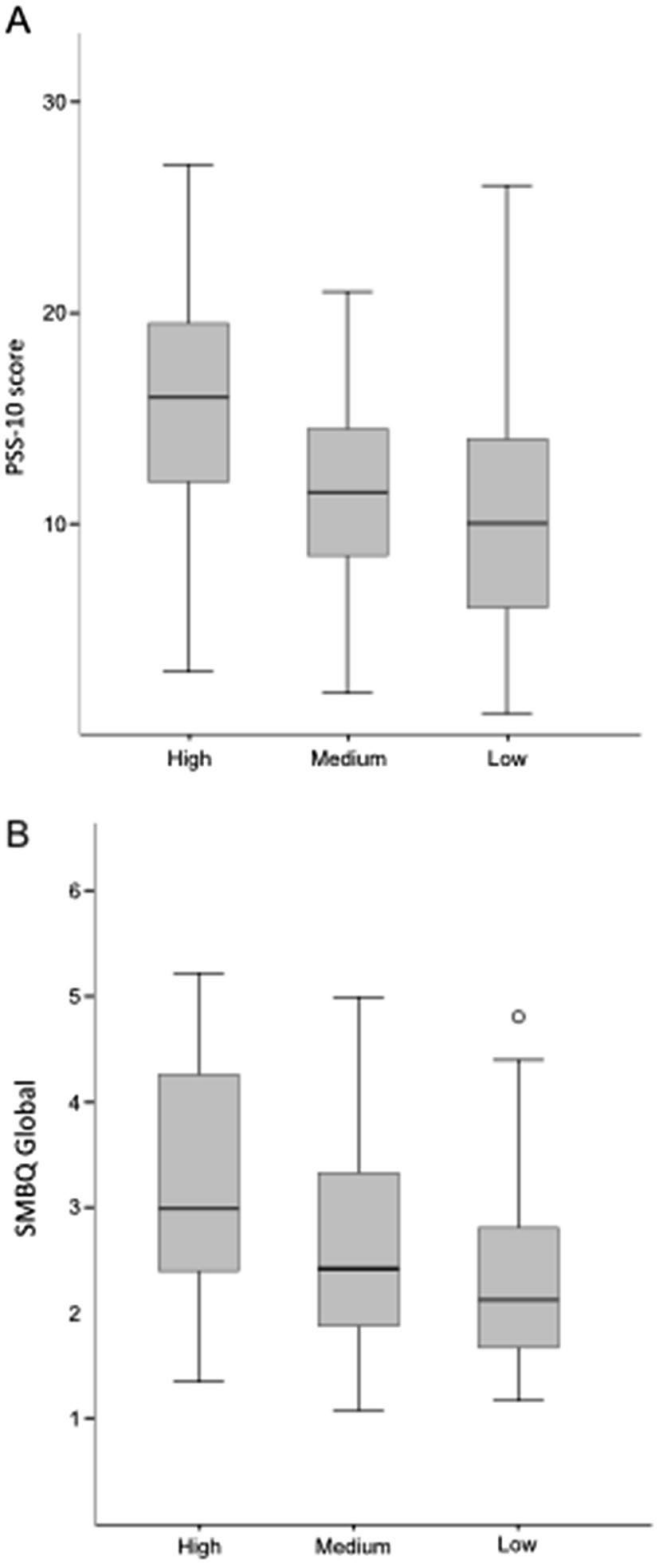
Box plot for PSS (**a**) and SMBQ (**b**) score for participants with different sensitivity to capsaicin. The difference between participants with high and low sensitivity is significant for both PSS and SMBQ. Whiskers represent quartiles and circles represent one participant

In Fig. 1a, b levels of PSS and SMBQ, respectively, are shown for the participants with different sensitivity to capsaicin. An ANOVA showed that there were significant differences between the groups; for PSS there was *F* (2.63) = 3.570, *p* = 0.029, for SMBQ there was *F* (2.63) = 3.359, *p* = 0.041. A post hoc test with Tukey’s HSD revealed differences between low and high capsaicin sensitivity in PSS (*p* = 0.027) and SMBQ (*p* = 0.032).

There were no significant differences in sensitivity to capsaicin between men and women or new and former patients (*p* = 0.594 and *p* = 0.655, respectively, Mann–Whitney *U* test). We also compared PSS and SMBQ for new and former patients and there were no differences (*p* = 0.56 for PSS and *p* = 0.41 for SMBQ).

Table 3 shows the number of participants who reported general and mucosal symptoms. Nobody did only report skin symptoms. There were 52 participants who reported skin symptoms often or sometimes, 61 who reported skin and mucosal symptoms often or sometimes and 62 participants who reported skin and general symptoms often or sometimes.

**Table 3:** Number of participants reporting symptoms often or sometimes during the last month

Symptom	Yes	No
Tiredness	57	8
Headache	53	12
Dry throat	52	13
Dry eyes	49	16
Throat irritation/hoarseness	49	16
Eye irritation	50	15
Cough	47	18
Nose dry/irritation	47	18
Stuffy nose	46	19
Heavy headiness	44	21
Difficulties to concentrate	37	28

## Discussion

In this study, we have found that participants that sometimes or often report itching, stinging, tight or burning facial skin and facial erythema due to exposure to a certain buildings, were significantly more sensitive to capsaicin. This is an interesting association since TRPV1 is an important mediator for itch and stimulation of the receptor also elicit a burning, stinging as well as vasodilation (Gouin et al. 2017). The results encourage search for TRPV1 agonists in the indoor environments where people report building-related skin symptoms.

Capsaicin has been used in several studies of Sensitive Skin (SS) to identify people with altered cutaneous nociception (Abbas 2020; Talagas et al. 2020). An expert group within the International Forum for the Study of Itch defined SS as “A syndrome defined by the occurrence of unpleasant sensations (stinging, burning, pain, pruritus and tingling sensations) in response to stimuli that normally should not provoke such sensations. These unpleasant sensations cannot be explained by lesions attributable to any skin disease. The skin can appear normal or be accompanied by erythema. Sensitive skin can affect all body locations, especially the face” (Misery et al. 2020). This description could also be applicable on participants in this study. The symptoms are similar, no skin disease explaining symptoms were found and some of the participants experienced erythema at their work places. Other similarities between SS and BRS are that females suffer from the symptoms more often than men and that facial symptoms are most common but other sites of the body may be involved (Norback 2009; Stenberg et al. 1993).

Factors associated with SS that has been identified are changes in transepidermal water loss (TEWL), thin stratum corneum, decreased hydration of stratum corneum, decreased lipids like ceramides and sphingolipids but, increased neutral lipids and, increased number of sweat glands. There have also been found alterations in neurosensory function like increased epidermal innervations, decrease in intraepidermal nerve fibre density (peptidergic C-fibres), heightened neurosensory input, upregulation in expression of TRPV1, and genetic variation in TRPV1 (Farage 2019). It remains to be investigated if these factors also are of importance for people with building-related skin symptoms.

It is interesting to note that different chemical substances can elicit SS symptoms and cosmetics is the most common trigger factor (Misery et al. 2020). Various chemicals have been used to study SS. In a study by Chen et al. (2018) they used lactic acid, dimethyl sulfoxide and sodium lauryl sulphate to study the symptoms and discuss various possible mechanisms based on their own and others’ results. In another study, lactic acid and capsaicin was used to identify people with SS and it was found that people may react to both lactic acid and capsaicin or just one of the agents (Sun et al. 2016). These two studies illustrate the complexity of SS which may also be applied for building-related skin symptoms.

Another association found in this study was that participants with high sensitivity to capsaicin also scored higher on scales for both short-term and long-term stress (PSS and SMBQ, respectively) compared with people with low sensitivity to capsaicin (see Fig. 1a, b). The relation between perceived stress and symptoms of skin diseases is well known (Alexopoulos and Chrousos 2016) as well as for BRS (Azuma et al. 2015; Bakke et al. 2007; Lu et al. 2018; Marmot et al. 2006). However, results from different studies are contradictory. Fukuda et al. (2015) found an association between psychological stress, measured as salivary α-amylase and chromogranin A, and impaired SC and recovery after tape striping. The result could not be confirmed in a similar study by Benham (2016) who used self-reported stress (PSS) to assess stress. One possible explanation for the different results could be a difference in severity of created skin damage between the studies. In the first study they used 15 tape strips while the second used up to 50 tape strips resulting in a deeper damage to the skin. In this study we found that participants who reacted to the lowest capsaicin concentrations scored higher on PSS and SMBQ which supports Fukuda’s findings. It is known that stress is often associated with BRS (Norback 2009) and an impaired skin barrier due to stress could be one of the mechanisms behind that relation by increasing sensitivity to chemicals in the air.

Another effect of stress, identified in mice, is an increased localized expression of TRPV1 (Ma et al. 2019; Zheng et al. 2015). If this is applicable to humans it may also contribute to the reason for participants with high capsaicin sensitivity scoring higher on PSS and SMBQ. Since TRPV1 is expressed on free nerve endings and keratinocytes in epidermis, an increased density of TRPV1 can result in higher sensitivity to chemicals entering the skin, resulting in itching, burning, stinging or tingling sensations as well as vasodilation. Further, in a study by Yun et al. (Yun et al. 2011) it was found that inhibition of TRPV1 increases recovery after tape striping in animal models. A prolonged stimulation of TRPV1 could affect SC and enhance penetration of extrinsic chemicals. This results might have implications for this study since the nature of the test, with symptoms recorded for a duration of only 3 min, indicates that it is defects in the barrier that are captured with the test. It is possible that an impaired barrier is a result of a vicious circle that leads to the skin symptoms related to BRS.

It is important to note that the average scores for PSS and SMBQ for the different groups are within normal levels, though individual people score higher. For PSS, average levels varied between 10.5 and 15.4 while the average score in the Swedish population is 13.96 ± 6.34 according to Nordin and Nordin (2013). When it comes to SMBQ a value greater than 4 is often used as a clinical threshold value for burnout and > 3.75 indicate long-term stress (Glise et al. 2014; Grossi et al. 2003) while we found average levels between 2.4 and 3.3 for the different groups.

A strength with the study is the close temporal proximity between capsaicin tests and the time when participants answered questions about stress. Another strength is the fact that participants in the group are homogenous. All of them worked at the same hospital and were medically examined and according to the physician, there were no other explanations for their symptoms than the indoor environment.

It is worth to underline that the group without skin symptoms were participants with general symptoms or mucosal symptoms (Table 3). We do not know if participants with skin symptoms had a less efficient skin barrier, if they had a higher local expression of TRPV1 or if there were any other reason for found difference between the groups. It would be interesting to compare TRPV1 expression and barrier function for people with building-related skin symptoms with both populations with BRS and without BRS.

## Conclusions

We found an association between sensitivity to capsaicin and facial skin symptoms among people with BRS. We also found an association between capsaicin sensitivity and perceived stress (both short-term and long-term stress). These findings encourage further research to find possible explanation for mechanisms of symptoms associated with BRS.

## Data Availability

Data are available upon request.

## References

[CR1] Abbas MA (2020). Modulation of TRPV1 channel function by natural products in the treatment of pain. Chem-Biol Interac.

[CR2] Alexopoulos A, Chrousos GP (2016). Stress-related skin disorders. Rev Endoc Metabol Dis.

[CR3] Azuma K, Uchiyama I, Katoh T, Ogata H, Arashidani K, Kunugita N (2015). Prevalence and characteristics of chemical intolerance: a Japanese population-based study. Arch Environ Occup Health.

[CR4] Bakke JV, Moen BE, Wieslander G, Norback D (2007). Gender and the physical and psychosocial work environments are related to indoor air symptoms. J Occup Environ Med.

[CR5] Benham G (2016). Skin barrier recovery is not associated with self-perceived stress. Stress Health.

[CR6] Candi E, Schmidt R, Melino G (2005). The cornified envelope: a model of cell death in the skin. Nat Rev Mol Cell Biol.

[CR7] Chen SY, Yin J, Wang XM, Liu YQ, Gao YR, Liu XP (2018). A new discussion of the cutaneous vascular reactivity in sensitive skin: a sub-group of SS?. Skin Res Technol.

[CR8] Choi E-H, Brown BE, Crumrine D, Chang S, Man M-Q, Elias PM, Feingold KR (2005). Mechanisms by which psychologic stress alters cutaneous permeability barrier homeostasis and stratum corneum integrity. J Invest Dermatol.

[CR9] Cohen S (1988). Perceived stress in a probability sample of the United States. The social psychology of health.

[CR10] Deng P-Y, Li Y-J (2005). Calcitonin gene-related peptide and hypertension. Peptides.

[CR11] Edvardsson B, Stenberg B, Bergdahl J, Eriksson N, Linden G, Widman L (2008). Medical and social prognoses of non-specific building-related symptoms (sick building syndrome): a follow-up study of patients previously referred to hospital. Intern Arc Occup Environ Health.

[CR12] Farage MA (2019). The prevalence of sensitive skin. Front Med.

[CR13] Fisk WJ, Mirer AG, Mendell MJ (2009). Quantitative relationship of sick building syndrome symptoms with ventilation rates. Indoor Air.

[CR14] Fukuda S, Baba S, Akasaka T (2015). Psychological stress has the potential to cause a decline in the epidermal permeability barrier function of the horny layer. Int J Cosmet Sci.

[CR15] Glas B, Stenberg B, Stenlund H, Sunesson AL (2015). Exposure to formaldehyde, nitrogen dioxide, ozone, and terpenes among office workers and associations with reported symptoms. Int Arch Occup Environ Health.

[CR16] Glise K, Ahlborg G, Jonsdottir IH (2014). Prevalence and course of somatic symptoms in patients with stress-related exhaustion: does sex or age matter. BMC Psychiatry.

[CR17] Gouin O (2017). TRPV1 and TRPA1 in cutaneous neurogenic and chronic inflammation: pro-inflammatory response induced by their activation and their sensitization. Protein Cell.

[CR18] Grossi G, Perski A, Evengard B, Blomkvist V, Orth-Gomer K (2003). Physiological correlates of burnout among women. J Psychosom Res.

[CR19] Hunter HJA, Momen SE, Kleyn CE (2015). The impact of psychosocial stress on healthy skin. Clin Exp Dermatol.

[CR20] Ige J (2018). The relationship between buildings and health: a systematic review. J Public Health Manage Prac.

[CR21] Jafferany M, Patel A (2019). Understanding psychocutaneous disease: psychosocial and psychoneuroimmunologic perspectives. Int J Dermatol.

[CR22] Jourdain R, Bastien P, De Lacharriere O, Rubinstein G (2005). Detection thresholds of capsaicin: a new test to assess facial skin neurosensitivity. J Cosmet Sci.

[CR23] Kemp PC, Dingle P, Neumeister HG (2004). Particulate matter intervention study: a causal factor of building-related symptoms in an older building. Indoor Air.

[CR24] Kezic S, Nielsen JB (2009). Absorption of chemicals through compromised skin. Int Arch Occup Environ Health.

[CR25] Lu CY, Tsai MC, Muo CH, Kuo YH, Sung FC, Wu CC (2018). Personal, psychosocial and environmental factors related to sick building syndrome in official employees of Taiwan. Intern J Environ Res Public Health.

[CR26] Ma W, Li L, Xing S (2019). PGE2/EP4 receptor and TRPV1 channel are involved in repeated restraint stress-induced prolongation of sensitization pain evoked by subsequent PGE2 challenge. Brain Res.

[CR27] Marmot AF, Eley J, Stafford M, Stansfeld SA, Warwick E, Marmot MG (2006). Building health: an epidemiological study of "sick building syndrome" in the Whitehall II study. Occup Environ Med.

[CR28] Melamed S, Kushnir T, Shirom A (1992). Burnout and risk factors for cardiovascular diseases. Behavior Med.

[CR29] Misery L (2020). Pathophysiology and management of sensitive skin: position paper from the special interest group on sensitive skin of the International Forum for the Study of Itch (IFSI). J Europ Academy Dermatol Venereol.

[CR30] Moore C, Gupta R, Jordt SE, Chen Y, Liedtke WB (2018). Regulation of pain and itch by TRP channels. Neurosci Bull.

[CR31] Norback D (2009). An update on sick building syndrome. Curr Opin Allergy Clin Immunol.

[CR32] Nordin M, Nordin S (2013). Psychometric evaluation and normative data of the Swedish version of the 10-item perceived stress scale. Scandinavian J Psych.

[CR33] O'Connor CM, Adams JU (2010) Essentials of cell biology NPG education. https://www.nature.com/scitable/ebooks/essentials-of-cell-biology-14749010/118241220#NATED_OUTLINE_1. Accessed May 15 2020

[CR34] Sauni R, Verbeek JH, Uitti J, Jauhiainen M, Kreiss K, Sigsgaard T (2015). Remediating buildings damaged by dampness and mould for preventing or reducing respiratory tract symptoms, infections and asthma. Cochrane Database Sys Rev.

[CR35] Skulberg KR, Skyberg K, Kruse K, Eduard W, Djupesland P, Levy F, Kjuus H (2004). The effect of cleaning on dust and the health of office workers—an intervention study. Epidemiology.

[CR36] Stenberg B, Mild KH, Sandstrom M, Sundell J, Wall S (1993). A prevalence study of the sick building syndrome (SBS) and facial skin symptoms in office workers. Indoor Air.

[CR37] Sun L, Wang X, Zhang Y, Wang T, Li X, Ma Y (2016). The evaluation of neural and vascular hyper-reactivity for sensitive skin. Skin Res Technol.

[CR38] Talagas M, Lebonvallet N, Berthod F, Misery L (2020). Lifting the veil on the keratinocyte contribution to cutaneous nociception protein. Cell.

[CR39] Wang SE (2017). TRPV1 regulates stress responses through HDAC2. Cell Rep.

[CR40] World Health Organization (1983) Indoor air pollutants : exposure and health effects : report on a WHO meeting, Nördlingen, 8–11 June 1982. EURO reports and studies, 78. Copenhagen

[CR41] Yun JW (2011). TRPV1 antagonist can suppress the atopic dermatitis-like symptoms by accelerating skin barrier recovery. J Dermatol Sci.

[CR42] Zheng G, Hong SS, Hayes JM, Wiley JW (2015). Chronic stress and peripheral pain: Evidence for distinct, region-specific changes in visceral and somatosensory pain regulatory pathways. Exp Neurol.

